# Study on Drive System of Hybrid Tree Harvester

**DOI:** 10.1155/2017/8636204

**Published:** 2017-05-28

**Authors:** Shen Rong-feng, Zhang Xiaozhen, Zhou Chengjun

**Affiliations:** School of Transportation and Civil Engineering, Fujian Agriculture and Forestry University, Fuzhou 350002, China

## Abstract

Hybrid tree harvester with a 60 kW diesel engine combined with a battery pile could be a “green” forest harvesting and transportation system. With the new design, the diesel engine maintains a constant engine speed, keeping fuel consumption low while charging the batteries that drive the forwarder. As an additional energy saving method, the electric motors work as generators to charge the battery pile when the vehicle moves downhill. The vehicle is equipped with six large wheels providing high clearance over uneven terrain while reducing ground pressure. Each wheel is driven via a hub gear by its own alternating current motor, and each of the three wheel pairs can be steered independently. The combination of the diesel engine and six electric motors provides plenty of power for heavy lifting and pulling. The main component parameters of the drive system are calculated and optimized with a set of dynamics and simulated with AVL Cruise software. The results provide practical insights for the fuel tree harvester and are helpful to reduce the structure and size of the tree harvester. Advantage Environment provides information about existing and future products designed to reduce environmental impacts.

## 1. Introduction

Although the tree harvester greatly improves the efficiency of the cutting tree, the tree harvester will have a negative impact on the surrounding environment [1]. For example, they will produce gas, vibration, noise, dust, oil, and gas. Taking gas as an example, forest harvesters mainly produce CO, HC, NOx, and solid particles that will cause the greenhouse effect, haze, photochemical smog pollution, and harm to human body and the environment. There is still a lot of work required to improve the design of forest harvesters to minimize environmental damage. In recent years, researchers and engineers started to notice the fact that harvesters consume large amount of diesel fuel and emit fumes and carbon dioxide, and fuel costs are substantial‎ [2, 3]. With increase in regulatory pressures for lower emissions and a growing demand for machines that cost less to operate, manufacturers started to look for new harvesters with electric and hybrid drives to replace the traditional hydraulic and mechanical driving system [4].

A harvester is a type of heavy forestry vehicle employed in cut-to-length logging operations for felling, delimbing, and bucking trees. A forest harvester is typically employed together with a forwarder that hauls the logs to a roadside landing. Harvesters are employed effectively in level to moderately steep terrain for clearcutting areas of forest. For very steep hills or for removing individual trees, humans working with chain saws are still preferred in some countries. In northern Europe, small and manoeuvrable harvesters are used for thinning operations; manual felling is typically only used in extreme conditions, where tree size exceeds the capacity of the harvester head, or by small woodlot owners.

A hybrid vehicle power system means that the vehicle power system uses two or more different types of power technology [5]. The Toyota FC Bus was developed by Toyota, based on the company's experience in developing FC buses together with Hino Motors, Ltd. The Toyota Fuel Cell System (TFCS), which was developed for the Mirai fuel cell vehicle (FCV), has been adopted to provide better energy efficiency in comparison with internal combustion engines and to deliver superior environmental performance with no CO2 emissions or substances of concern (SOCs) when driving ‎[6]. The John Deere 644K Hybrid hybrid-electric powertrain with reduced mechanical complexity delivers amazing responsiveness for efficient stockpiling, ramp climbing, and truck loading. And it consumes an average of 25% less fuel ‎[7]. The 336E Hybrid is the industry's first hydraulic hybrid excavator. This unique machine uses recovered energy from the swing to load your trucks all-day long using up to 33 percent less fuel ‎[8]. In addition to the greater power transmission efficiency achieved by adopting a hybrid drive train, the Hitachi ZW220HYB-5B reduces energy losses in the hydraulics and delivers a more appropriate output when digging. As a result, the new model achieved a 31% reduction in fuel‎ [9]. The Komatsu HB215LC-2 third-generation hybrid excavator dependable and durable components harness free kinetic energy, convert it to electricity for a powerful and quiet performance, and help to reduce carbon footprint and fuel consumption by up to 40%‎ [10]. The Cross Coupe GTE vehicle comes with five driving profiles. Ponsse and Elforest Technologies have jointly studied 6 and 4 hybrid technology tree harvesters, which are more economical and more efficient than traditional harvesting machines [11]. The Mercedes-Benz F 015 Luxury electric hybrid system has a total range of 1,100 kilometers, including around 200 kilometers of battery-powered driving and around 900 kilometers on the electricity from the fuel cell ‎[12]. Elforest Technologies and Ponsse have developed a hybrid electric prototype with Elforest Technologies' “Electric Turbo.” The “Electric Turbo” is installed on a Ponsse Ergo harvester and is now performing field tests‎ [13]. Hybrid power harvester is the core of hybrid power harvester study; the arrangement, selection, and parameters of structure have an important influence on harvester. It needs reasonable design to ensure the power and economy of the whole harvester. To improve vehicle dynamics and energy efficiency, a new type of hybrid power harvester transmission was put forward. The key components of the hybrid power transmission were designed and verified so as to achieve the rational design of the structure. The different structure proposals of diesel-electric hybrid harvester transmission were raised firstly; the most reasonable structure of transmission was determined. Then parameters match was done according to the dynamic performance. Finally, the match result was verified by means of computer simulation.

## 2. System Design Parameters of Hybrid Harvester Transmission

Harvester has short distance about its motion. The general working scope is forest and the harvester speed can reach 2–8 km h^−1^ and the climbing degree is about 35° [14]. The harvester working in southern China mainly is the artificial forest. For Southern China, terrain is rugged; the harvester needs to have enough power ‎[15–17]. Taking CFJ20 harvester as the design blueprint, the basic parameters and performance indicators of CFJ20H hybrid power harvester were shown in Table 1.

Harvester is heavy vehicle; a series hybrid electric drive system is more appropriate. In order to ensure stable operation and good energy saving effect, the key components study of transmission was needed. Currently, the design method of hybrid power system parameters was that the dynamic equation of the vehicle was calculated first; then the vehicle parameters and performance indicators were set. Followed by CFJ20H hybrid power harvester's calculation of the kinetic equation, the power system parameters were got ‎[18].

### 2.1. The Matching of Motor Parameters and Methods

A series hybrid harvester was driven by the motor directly; therefore the power must meet the nominal demand. As for harvester, the main consideration was two working environments, road and woodland. The basic parameters of CFJ20H hybrid harvester were shown in Table 2. Since the maximum speed of hybrid harvester is not less than 50 km h^−1^, the grade ability is up to 60% at least ‎[19]. So the maximum power of the hybrid harvester was calculated according to dynamic performance indicators. And the larger one was taken for the selection of motor.

As for a series hybrid vehicle, motor-driven rated power depends entirely on automotive acceleration and performance requirements for motor characteristics and transmission characteristics. So the driving power of the hybrid harvester was composed of rolling resistance, air resistance, and air resistance power‎ [20].

Rated power value of motor is

(1) $$\begin{array}{c} P_{f}=\frac{\delta M}{2t}\left(\;V_{f}^{2}+V_{i}^{2}\;\right)+\frac{2}{3}Mgf_{r}V_{f}+\frac{1}{5}\rho_{\alpha }C_{x}AV_{f}^{3} \end{array}$$

and average power of motor is

(2) $$\begin{array}{c} P_{e}=\frac{2}{3}Mgf_{r}V_{f}+\frac{1}{5}\rho_{\alpha }C_{x}AV_{f}^{3}, \end{array}$$

where *δ* is inertia coefficient (1), and because of the small weight, it is ignored; *M* is weight of harvester (kg); *V*_*i*_ is basic speed (km h^−1^); *t* is acceleration time, s; *C*_*x*_ is drag coefficient, 1.1; *g* is acceleration of gravity, 9.81 m s^−2^; *f*_*r*_ is rolling resistance; *V*_*f*_ is final velocity, km h^−1^; *ρ*_*α*_ is air density, 1.23 kg/m^3^; *A* is frontal area, 5 m^2^.

According to the above formula, the maximum power and average power of the land and woodland were calculated, which were shown in Table 3.

According to Table 3, the maximum speed of the motor is more than 50 km/h and the minimum power is more than 54 KW. Because of the low speed and the fact that the hybrid power harvester belongs to heavy machinery with large quality, it needs more power. Therefore, LJEV permanent magnet motor was selected. The structure of the motor is simple, which is low-speed high torque performance, so it is suitable for hybrid power harvester; the specific parameters were shown in Table 4.

### 2.2. Matching and Method of Transmission

The design of transmission should meet the need that while the motor is at the highest speed, the harvester is at maximum speed.

(3) $$\begin{array}{c} i=\frac{\pi n_{max}r}{30V_{max}}, \end{array}$$

where *i* is drive ratio of transmission; *n*_max_ is the maximum speed of motor, r/min; *r* is radius of the wheel, 0.6665 m; *V*_max_ is maximum speed, m/s.

Through calculation, the maximum transmission ratio in road is 15 and is 70 in woodland. The transmission ratio in road is lower than that in woodland. The transmission of main reducer is 5. The reducer has 4 gears, so a gear ratio of reducer should meet the climbing performance in woodland. The maximum ratio is 70/5 = 14. And the minimum ratio is 15/5 = 3. The ratio of two transmissions is not more than 1.7~1.8. So the ratio of each block transmission reducer was 14, 9, 5, 3.

### 2.3. Parameter Matching and Method of Battery Packs

The battery is one of the sources of normal driving energy of hybrid power harvester; its performance directly affects the performance of the harvester ‎[21]. Battery required continuous discharge-charge-discharge, so the battery is relatively high. Currently, hybrid vehicles are widely using lead-acid batteries, nickel metal hydride batteries, and lithium batteries. Lithium batteries have greater specific energy, specific power, and life; at the same time, their weight is small. So the lithium battery was selected for storage system of hybrid power harvester. In order to take full advantage of the power capacity of the traction motor, the total power of battery pack should be not less than rated power of motor ‎[22]. According to the motor parameter, the motor voltage was selected as 320 V and the rated power of motor is 75 KW.

Under the battery pack rated voltage, the amount of single-cell battery of hybrid power harvester was calculated:

(4) $$\begin{array}{c} n_{c}=\frac{U_{B}}{u}=\frac{320}{12}=26.7\left(\;\text{one}\;\right)\;\left(\text{take as }27\right), \end{array}$$

where *n*_*c*_ is the number of single-cell battery series; *u* is the rated voltage of single-cell battery (a single-cell lithium battery is 12 V).

According to motor power, battery power is obtained:

(5) $$\begin{array}{c} p_{b}\geq \frac{p_{f}}{\eta }=\frac{100}{0.95}=105\;KW, \end{array}$$

where *p*_*b*_ is power of battery; *p*_*f*_ is peak power of motor; *η* is motor efficiency.

Maximum current of battery is

(6) $$\begin{array}{c} I_{max}=\frac{p_{b}}{U_{B}}=\frac{105\times 1000}{320}=328\;A. \end{array}$$

The number of batteries in parallel is

(7) $$\begin{array}{c} n_{b}=\frac{I_{max}}{C}=\frac{328}{70}=4.7\;\left(\text{take to be }5\right), \end{array}$$

where *n*_*b*_ is the number of batteries in parallel; *C* is battery capacity.

Considering the above battery parameters, the final choice is model CN/12 100 150 lithium battery from Electronic Technology Co., Dongguan City. The parameters of the single-cell battery were shown in Table 5.

### 2.4. CFJ20H Hybrid Power Harvester Model

**Figure 1:**
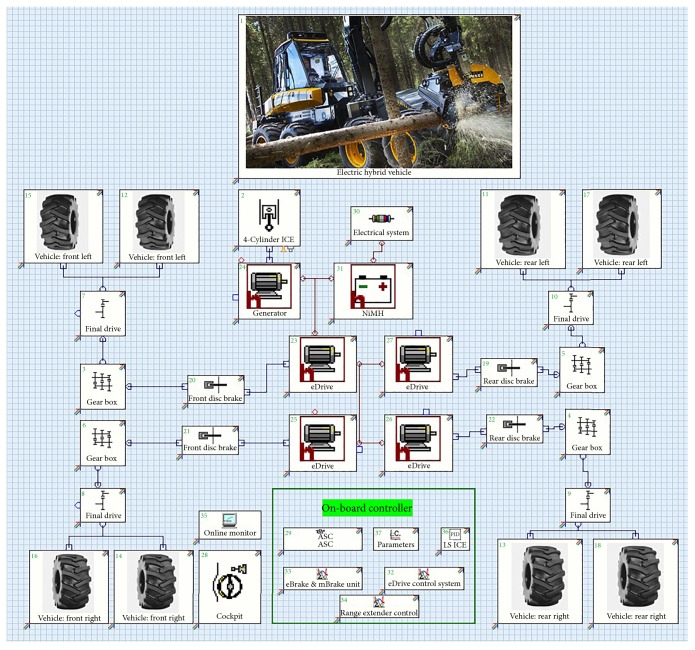
Model of CFJ20H hybrid power harvester.

Applying for AVL Cruise, the simulation of hybrid power harvester was made. The interface and a large number of computing tasks of software are a good choice for modeling and simulation. In the whole design process, it can greatly reduce manpower and material resources, shorten the time, and so forth. The object is a series hybrid power harvester; its transmission is shown in Figure 1. The task of the AVL complex program is to solve the industry in the mechanical domain, which can calculate the components of all nodes and units and the interaction between them and the control system ‎[23]. According to the operation of the logger, use the software to set the operating conditions for the “run Working + cycle Driving.” Through the Cruise AVL software, the simulation analysis of the hybrid harvester can directly reflect the performance of the design of hybrid harvester and meet the reservation function requirement of the design.

## 3. Simulation Result

**Figure 2:**
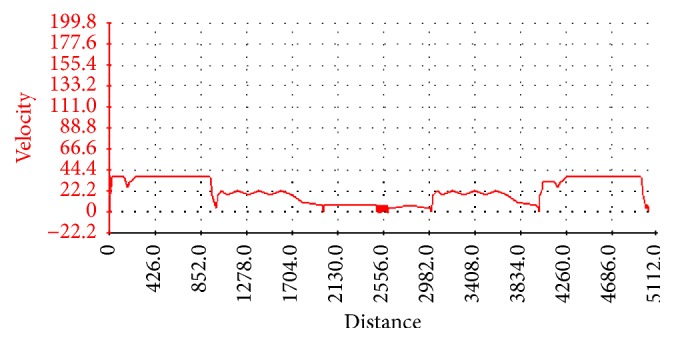
Operating condition.

Depending on the operation environment, the operating condition was determined. On the basis of analysis, the dynamic and economic performance of harvester was put forward. The work environment of harvester was road and woodland and most of time was in the forest. The operation condition of working woodland of felling machine is considered in this paper. AVL has complex programs; its task is solving the automotive sector of vehicle industry. The complex can calculate all nodes and elements of vehicle and their interaction and control systems. Forest operating condition is a special area. Because there is no mountain operating conditions, use “run Working + cycle Driving” conditions for the simulation of the tree harvester running cycle, as shown in Figure 2.

Through the simulation analysis of the harvester by AVL Cruise software, it can directly reflect the performance of the designed hybrid harvester and satisfy the design requirements. The result was shown in Table 6. It is shown that the design of hybrid power harvester can meet the design requirement. The maximum speed and maximum climbing degree were fairly with the design requirement, while the acceleration time of 0–50 km h^−1^ was less than the design requirement. The fuel consumption of hundred kilometers was less than design requirement. So the hybrid power harvester designed meets the requirement.

### 3.1. Climbing Performance

**Figure 3:**
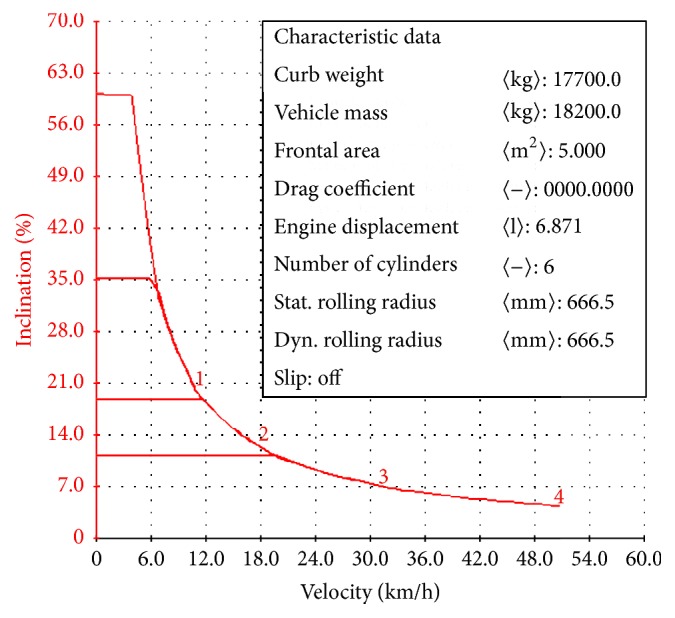
Climbing degree of hybrid power harvester at full load.

The gradeability of hybrid harvester at full load was shown in Figure 3. As the figure shows, the climbing degree of harvester is 60.04%, which meets the requirement. According to the relationship of gradeability, weight, rolling resistance, and air resistance, the climbing performance of hybrid harvester is inversely proportional to its own weight and resistance, and the relationship with traction force is directly in proportion. So the hybrid power machine can improve climbing performance in no-land.

### 3.2. Maximum Speed

As Table 7 shows, the maximum speed of the harvester was increased with the transmission range increasing. Because the drive ratio is relatively small, according to formula of the maximum speed, the drive ratio and the wheel radius are constant. The maximum speed is inversely proportional to the transmission ratio of the gearbox with the rotational speed. So while the transmission ratio is smaller and the rotating speed is higher, the maximum speed of hybrid power harvester is higher.

### 3.3. Acceleration

**Figure 4:**
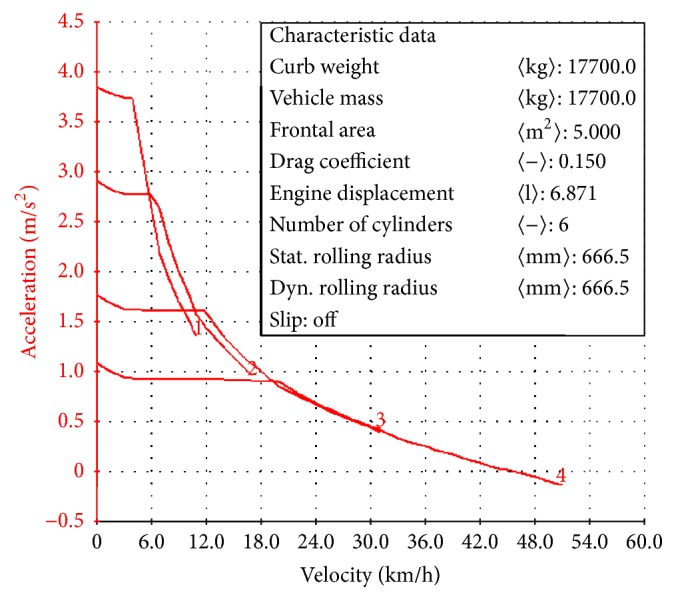
The acceleration and speed graph of hybrid.

The relationship of acceleration and speed of the harvester was shown in Figure 4. As a whole, the speed is higher, and the acceleration is lower. Two adjacent acceleration curves have intersection point. Apparently, in order to obtain the shortest acceleration time, it should be switched to the high grade from low grade at the point of speed. It was shown in Figure 4 that the maximum speed is 3.85 km h^−1^.

**Figure 5:**
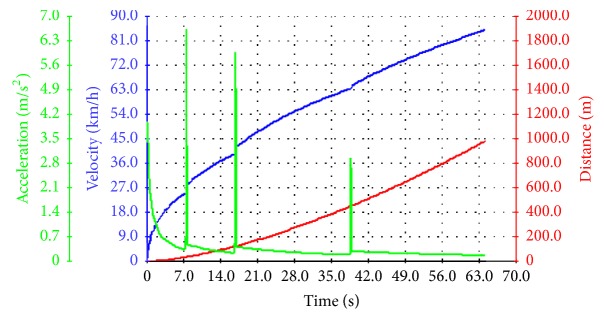
Start continuous shift acceleration curve.

As Figure 5 shows, the acceleration time of 0–50 km h^−1^ is 16.22 s, which meets the design requirements of vehicle acceleration performance. The shift mode was set in the maximum acceleration in the Cruise software. Speed and distance will increase with time gradually increasing, and the speed in front accelerates faster and the rear is eased. The change of acceleration with the downward trend can be seen from the acceleration-time curve. Because the hybrid power harvester starts and changes gear continuously, acceleration will decrease with the gear increase. There is a momentary interruption in the process of shifting; it takes time to shift gear. In order to improve acceleration performance, reducing shifting time can improve power performance of harvester.

## 4. Summary

Interest in hybrid vehicles for forestry is increasing and we can be sure that some type of hybrid harvester will be running in forest in the next few years. Such applications might include an electrically powered harvester. When running at slow speeds or with low grain flow, these components would operate at a slower speed. As the harvester speeds up, the components would pick up proportionate speed. Items like unloading augers could also be powered with electric motors to provide a steady increase in speed, compared to sudden shock starts on existing systems. The research describes four parts, structural scheme of transmission, dynamics and economic indicators, parameters matching of transmission, and simulation and analysis of hybrid power harvester transmission. First, the structure scheme of hybrid power harvester was determined. Dynamics and economy indicators were put forward to powertrain. Parameter design and selection were made to main components of transmission, which include motor, batteries, generators, and engines. Applying for AVL Cruise, the transmission system was verified. The result shows that the economy and dynamic performance of hybrid power harvester can meet the design requirement; thus the transmission design is reasonable.

## Figures and Tables

**Table 1:** The basic parameters and performance indicators of CFJ20H hybrid power harvester.

Category	Parameter name	Numerical
Basic parameters	Curb weight (kg)	17700
	Maximum weight (kg)	18200
	Wheelbase (mm)	4000
	Frontal area (m^2^)	5
	Air resistance coefficient	1.1
	Woodland rolling resistance coefficient	0.15
	Terrestrial rolling resistance coefficient	0.015
	Number of wheels	8
	Diameter of the tire (mm)	1333
Performance parameters	Maximum speed (km h^−1^)	50
	0–50 km h^−1^ acceleration time (s)	≤23
	Maximum grade	≥31° (60%)
	Fuel economy	≤23 L/100 km

**Table 2:** Basic parameters of the hybrid power harvester.

Operating environment	Acceleration time (s)	Maximum speed (km/h)	Load	Rolling resistance	Weight (kg)
Road	30	50	No-land	0.015	17700
Woodland	5	10	land	0.15	18200

**Table 3:** Total motor power of CFJ20H hybrid power harvester.

Operating environment	Maximum speed (km h^−1^)	Rated power of motor (KW)	Average power of motor (KW)
Terrestrial	50	83	28
Woodland	10	70	54

**Table 4:** Performance parameters of motor.

Index	Motor type	Rated voltage	Rated power	Peak power	Rated torque	Peak torque	Max. speed	Efficiency
Parameter value	LJEV	320 V	60 KW	100 KW	200 Nm	350 Nm	3000 rpm	More than 95%

**Table 5:** Single-cell lithium battery parameters.

Item	Battery type	Battery capacity (AH)	Rated voltage	Number	Weight of single-cell battery	Cycle life
Parameter value	CN/12100150	70	12 V	27 × 5	8.2 kg	2000 times

**Table 6:** Simulation result of dynamics.

Index	Simulation result	Design requirement
Maximum speed	50 km h^−1^	50 km h^−1^
Acceleration time of 0–50 km h^−1^	16.22 s	≤23 s
Maximum grade	60.04%	≥31° (60%)
Fuel consumption for one hundred kilometers (L/100 km)	16.90	≤23 L/100 km

**Table 7:** The maximum speed of each gear.

Item	Parameter value			
Gear	1	2	3	4
The maximum speed (km h^−1^)	10	16	30	50
Rotating speed (r/min)	2785.91	2865.51	2984.90	2984.90

## Nomenclature

*δ*: Inertia coefficient (1): because of the small weight, it is ignored
*M*: Weight of harvester, kg
*V*_*i*_: Basic speed, 0 km h^−1^
*t*: Acceleration time, s
*C*_*x*_: Drag coefficient, 1.1
*g*: Acceleration of gravity, 9.81 m/s^2^
*f*_*r*_: Rolling resistance
*V*_*f*_: Final velocity, km h^−1^
*ρ*_*α*_: Air density, 1.23 kg/m^3^
*A*: Frontal area, 5 m^2^
*i*: Drive ratio of transmission
*n*_*r*_: The minimum speed of motor, r/min
*r*: Radius of the wheel, 0.6665 m
*V*_*r*_: Maximum speed, m/s
*n*_*c*_: The number of single-cell battery series
*u*: The rated voltage of single-cell battery (a single-cell lithium battery is 12 V)
*n*_*b*_: The number of batteries in parallel
*C*: Battery capacity.
